# 

**Published:** 1887-09

**Authors:** 


### CONTENTS SEPT., 1887, VOL. 3, NO. 3.

Original Communications.—A Case of Traumatic Tetanus successfully Treated by Hot Water Baths, T. J. Bennett, M. D. .... ........ 76

Cocaine in Venereal Surgery, A. A. Lyon, M. D .......... 77

Hernia, W. W. Walker, M. D .......... 81

Hadra's Operation for Cystocele, F. H. Tucker, M. D .... 82

A Rare Case of Dislocation, C. M. Harrison, M. D ...... 83

Society Notes.—T. S. M. A. Correction .......... 84

Austin District Medical Association .......... 85

Death of Dr. Wilson, and Resolutions Runnels County Medical Society .......... 86

Ninth International Medical Congress .......... 87

T. S. M. A. Appointments .......... 88

[illegible]p[illegible]ondence.—Letter from Washington—Ninth Interna-national Medical Congress .......... 89

Editorial.—The State Lunatic Asylum Trouble .......... 91

Still at It—The *N. Y. Medical Record* .......... 94

Editorial Supplement—A Roaring Farce .......... 1-3

Bibliography.—Transactions T. S. M. A .......... 95

Unwise Laws .......... 103

Drug Eruptions .......... 104

"Anaemia," Physician's Dose Book—Oxygen in Therapeutics .......... 105

Elementary Microscopic Technology .......... 106

Medical News and Miscellany.—Numerous Paragraphs, Wise and Otherwise .......... 107

Advertisers' Notices .......... 115

